# Aging Living at Home: Residential Satisfaction among Active Older Adults Based on the Perceived Home Model

**DOI:** 10.3390/ijerph18178959

**Published:** 2021-08-25

**Authors:** Josue G. Amián, David Alarcón, Cristina Fernández-Portero, Jose A. Sánchez-Medina

**Affiliations:** Department of Social Anthropology, Psychology and Public Health, Pablo de Olavide University, 41013 Sevilla, Spain; jgarami@upo.es (J.G.A.); dalarub@upo.es (D.A.); jasanmed@upo.es (J.A.S.-M.)

**Keywords:** older adult, perceived home, residential satisfaction

## Abstract

Background: Housing plays an important role in the aging process and health. The house and its nearby environment host most of the daily activities of older adults. Residential satisfaction (RS) has been relegated to physical issues such as accessibility. However, RS is also constituted by older adults’ perceptions about housing. This study analyzes the perceived home that develops RS in older adults. Methods: A random sample of 714 participants aged 50 to 84 (mean = 65; SD = 0.98) were used. Participants lived in ordinary housing in southern Spain. Scales measure perceptions of RS, meaning, functionality and belief of control over the home. Results: Analyses were performed using structural equation models to evaluate the dependence relationships between the different perceptions evaluated. We observe a direct influence of internal control on usability (β = 0.84) and perceived meanings (β = 0.49). However, external control shows a negative influence on the meaning of home (β = −0.14). Perceived usability (β = 0.68) and meaning (β = 0.32) positively influence RS. Conclusion: Perceptions of meaning, functionality and RS itself depend on internal housing-related control beliefs. Active older adults with higher internal control perceived their home fit better to the need of everyday life and improve RS.

## 1. Introduction

The role that housing plays in the aging process and health is widely recognized [1,2]. Several studies [3,4,5] indicate a complex interplay between housing and health in older adults. These studies show the objective and subjective aspect of the housing, (arquitectonic–environmental barriers or perceptions), are related to mobility problems, depressive symptoms and life satisfaction. Older adults who perceive a good home accessibility tend to achieve higher wellbeing and fewer depressive symptoms [3].

The house and its nearby environment host most of the daily activities of older adults, and this circumstance increases progressively with age. In old age, people spend more time at home, making it the anchor point of their activities. Therefore, housing is a central element in the daily life of older adults [6,7,8,9]. In this sense, it is very important to know which factors influence older adults’ satisfaction with their home. The objective of this study is to analyze the factors that develop the perception of residential satisfaction (RS) in active older adults.

Traditionally, the study of RS has been relegated to physical issues such as accessibility that have captured most of the attention and initiatives aimed at providing older adults with adequate housing [4,10,11]. However, RS is a more complex concept than solving functional or accessibility issues [12]. Related RS to accessibility means reducing this satisfaction to the mere resolution of impediments or architectural obstacles in the house itself. In this way, the social and psychological processes related to the physical space of the house are not considered. In this sense, the concept of “home” represents these psychological aspects as opposed to the concept of “house”. House refers to a structure of a residential space, a building, a house, rooms, etc. where people live. However, home is a symbolic place built with personal meanings that change over time [13]. Research that focuses on the objective and physical characteristics of housing forgets the importance of perceived experiences in the relationship of the home and well-being in old age [14,15,16]. Thus, satisfaction with housing is generated through important psychological links and identification between the person and the home. Knowledge of the physical spaces inside the house ceases to play a major role in the formation of RS. From this point of view, RS is constituted by the subjective perceptions that older people have about housing [7,9].

Oswald, Schilling, Wahl, Funge, Sixsmith and Iwarson [17] underscore a psychological perspective of the home environment. This study was to examine the home environment and its importance in healthy aging. The participants were 1223 older adults aged (80–89 years) and living alone in their private homes in Swedish, British and German urban regions. The result showed that it is the subjective experiences from living in the home itself that constitute what has been called perceived home (PH). The exploratory and confirmatory factor analysis and a structural equation model give empirical support to prove the model of four domains of PH: RS itself, home functionality, meaning and perception of control over the home itself. In addition, several studies [2,17,18] indicate that there is a close relationship between the different domains of PH. As a result, people who obtain a high RS score obtain high scores in perception of functionality, meaning and control over the home itself. The results of these studies indicate that people who have a high PH score develop effective links to their home environment and value home as support for their independent and autonomous life. Therefore, from the psychological perspective offered by PH, home is not only objective and behavioral aspects but also incorporates subjective, cognitive and emotional aspects [19].

As mentioned above, PH is composed of four perceptions: perception of satisfaction, functionality, meaning and housing control belief (HCB). The perception of home functionality focuses on the relationship between the activities that the person and the home can perform, both in the present and in the future. That is, they are the perceived possibilities of being able to develop in the house both the necessary and preferred activities that comprise the routines of daily life [20,21]. The domain of perceived functionality includes elements such as the functional capacity of the person, his/her adaptive and motivational strategies to develop certain activities, the physical barriers of housing and surroundings, and the repertoire of activities developed by a person in his/her home.

Older adults build the perceived meanings of home. Older adults build, in relation to their own home, symbolic representations of space and place. Home is not only a place where objective needs such as protection, support and access are met but also symbolic representations of space and place in relation to their own home represents individual meanings linked to the user’s experience and personality [15]. The meaning of the perceived home includes elements such as meaningful habits for the person, social contacts, assessments, goals, values, cognitions and emotions of a person regarding their home. That is, the meaning of home for a person describes a wide range of attachment processes that are generated when they develop affective, cognitive, behavioral and social bonds with their home.

HCB is the tension that occurs between what the person wants to do in the house and the pressures of the environment that prevent him/her from carrying them out [13]. The perception of HCB tries to analyze the explanations that older adults give about the events that happen in their home. These events can be explained based on internal control over them, derived from their own behavior, or they can be explained by external control exercised by other people, luck, destiny, etc.

Several studies [5,22] have shown that perceptions of HCB are closely related to maintaining autonomy and independence in everyday life and become increasingly important in old age. From the perspective of environmental gerontology [13,23], this autonomy and independence constitute the concept of competence, that is, what older people can do at home and that depends on them. In contrast, there are several demands that converge in the house itself, called the environmental press [24]. A good fit between a person’s competencies and the demands of the environment is a particularly important challenge in the aging process [25,26]. It should be noted that these adjustments between competencies and demands become very unstable because the capacity to adjust may vary ostensibly linked to the aging process itself [7]. Thus, the perceived HCB in the field of home is the result of the tension that is generated between competition and demand [27,28].

Perceptions of HCB offer significant theoretical potential, providing a conceptual link between PE fit and older adults [22], focuses on the interaction between characteristics of individual and environment. From this perspective, older adults become agents of change in their residential environment. The perception of control recognizes the proactive role of older adults, focusing directly on their agency as an engine of change that can determine not only physical changes in the home but also changes in the rest domains of PH [9].

We propose that RS is not another element within the perceptions of home but is built on perceptions of functionality, meaning and control over home. It is the final product that develops through the relationship with the domains of PH. The main objective of this study is to analyze how RS is developed from the relationships established between the different domains of PH using a structural equation model. This proposal would incorporate into Oswald’s studies an explanatory model of the role of each PH in the formation of RS.

The hypothesis is that the perception of HCB influences the perception of functionality and meaning of home. Perceived RS would be the final product of the previous PH.

## 2. Materials and Methods

### 2.1. Participants

The sample consisted of 714 older adults who voluntarily participated in the research. For the recruitment of the participants an informative talk was held on the objectives of the study and its ethical considerations. Those subjects who voluntarily decided to participate in the study signed the informed consent. All participants were active older adults over 50 years old without dependency and were autonomous. This criterion was employed because we aim to study the RS in active older adults. As recommended by SHARE project (Survey of Health, Ageing and Retirement in Europe) to study ageing in place, older adults were considered those people over 50 years of age [29]. That is, those who are in the retirement process and those who are in the process of research retirement and are planning and making decisions on different aspects related to their home [29,30].

### 2.2. Instrument

The housing options for older people (HOOP) questionnaire [31] is a nine-item tool scored on a Likert-type scale ranging from 1 (definitely not) to 5 (yes definitely). The HOOP questionnaire evaluates the satisfaction of older people with different aspects of their house and how they live in it. The scale is divided into three factors: satisfaction with the physical characteristics of the home (items 1–4), satisfaction with home security (items 5–7) and satisfaction with the propensity to change (items 8 and 9). A higher score indicates better perceived satisfaction with the dwelling itself. The reliability index of the scale (α = 0.84) and the internal consistency (KMO = 0.908) were very high.

Usability in My Home Questionnaire (UIMH) [27] were developed as a Likert type of sixteen items based on the degree to which the physical home environment supports the performance of activities at home. It is scored on a Likert-type response scale of 1 (not at all) to 5 (fully agree). It is divided into three factors: physical usability (items 1–4), relational usability (items 5–8) and usability of spaces (items 9–16). A higher score represents a better perception of usability. The scale has a high reliability index (α = 0.91) and internal consistency (KMO = 0.945).

The Meaning of Home Questionnaire (MOH) [32,33] includes 28 items to assess the subjective perceptions of older people about the meaning of their own home. It is divided into five factors: physical (items 1,3,6,7,12,13,25), behavioral (items 8,10,15,20,21,26), cognitive (items 2,9,16,17,22), emotional (items 4,14,18,23,27) and social (items 5,11,19,24,28). The questionnaire is scored on a Likert-type scale from 1 (strongly disagree) to 5 (strongly agree). High scores indicate a greater perceived meaning of home. The scale has a high reliability index (α = 0.77) and internal consistency (KMO = 0.901).

The housing-related control beliefs questionnaire (HCB) [22] is a scale of control over the home itself of 24 items with 3 factors: internal control (items 1,4,7,10,13,16,19), external control: others (items 2,5,8,11,14,17,20,23) and external control: luck (items, 3,6,9,12,15,18,21,24). Internal control indicates how control beliefs about the home depend on the person and the external control, others, means that other people around the old person make decisions about their houses and finally, external control, luck, indicates that control over the housing is due to random factors such as luck. The reliability index (α = 0.77) and the internal consistency (KMO = 0.796) of the scale were high.

### 2.3. Procedure

Data were collected in educational centers for older people, universities of the Third Age and senior associations. The data collection period was 6 weeks. The selection of the sample was intentional through the agreements signed with the University of Third Age of the Pablo the Olavide and Centers of seniors. Previous appointments were made for the collection of data. The questionnaires were administered by two research firms specializing in the administration and collection of data.

Data collection was applied in a collective manner in the centers. The researchers previously reported on the objectives of the study and asked older people to fill out the questionnaire and ask any questions they had about it. In any case, the time session for the questionnaire did not exceed 40 min, including rest time between questionnaires, which avoids the fatigue effect of older adults when taking psychological tests.

### 2.4. Data Analysis

The data analysis strategy was descriptive for the identification of the sociodemographic characteristics of the sample and the comparison of means and correlations for the analysis of the relationship between domains of the PH. Finally, analyses were performed using structural equation models to evaluate the dependence relationships between the different subjective perceptions evaluated. Data analysis was performed with SPSS (version 24, SPSS Inc., Chicago, IL, USA) and STATA (version 16, College Station, TX, USA). As multivariate normality distribution of the data, Mardia’s coefficient, was statistically significant (*p* < 0.05), the Satorra–Bentler correction of chi-square and standard errors was used and robust versions of CFI, TLI and RMSEA fit indices.

## 3. Results

### 3.1. Socio-Demographics Charaterristics

Table 1 shows the sociodemographic characteristics of the sample. Thus, the data indicate that 72.4% are women whilst 27.6% are men, which corresponds to the population representation of older people. In addition, these percentages of participation are representative of the group of adults who participate in programs, centers or associations, with women being the ones most likely to participate in social activities. If we consider the educational level, the majority of participants have primary studies (54.1%) or without studies (38.5%), compared to 7.5% with higher studies This fact also corresponds to the current population of older people who have less training or formal education than the current adult generations who will be part of the aging group in one or two decades.

Regarding age, the data indicate that 10.7% are between 50 and 59 years old, 49.6% are between 60 and 69 years, 31.5% are between 70 and 79 years old, and 8.2% are 80 or older. In addition, 77.2% of the interviewees lived together, compared to 22.8% who lived alone.

Furthermore, the majority of the older subjects in the study owned their own home (94.7%), compared to the 5.3% that rented. These data are significant and correspond to the housing policies of the Spanish state, where the population prefers to invest in housing rather than rent it, as is the case in other European Union countries.

Table 1 shows the housing size of the older people who participated in the study; 32.7% of them live in houses with less than 90 m^2^, 30.2% live in houses that have 90 to 120 m^2^, and 37.1% reside in homes with more than 120 m^2^. Finally, 22.8 live alone and 77.2 live accompanied; 55.3% of housing is located in urban areas and 44.68% in rural areas.

### 3.2. Descriptive Analysis of the Domains of PH

To study the PH of the active older adult a descriptive analysis was performed. The Usability in My Home Questionnaire (UIMH), Housing options for Older People (HOOP), the Meaning of Home (MOH) and housing-related control beliefs (HCB) questionnaires provide an overview of the perceptions about the adaptation of housing to the current needs of the older adult. Table 2 shows that there is a high perception of the usability of the house (mean = 4.22, SD = 0.76); there is a very high satisfaction of the participants with housing (mean = 4.06, SD = 0.78); and the meaning of the house for older people corresponds to a secure site, well adapted to the needs of the older people, comfortable, intimate, cozy, relaxing and where they do what they fancy (mean = 3.69 y SD = 0.46). Finally, the perceived control of their home is moderate since the different items are divided into mutually exclusive factors (mean = 3.20 y SD = 0.57). A multivariate MANOVA was performed with the scale measures as dependent variables and gender by age group as independent factors, non-significant differences were found for the principal and interaction effects (*p* > 0.05)

Table 3 shows a detailed analysis of the factors of each scale. For the UIMH questionnaire, the highest mean is obtained by the relational functionality factor (mean = 4.34 and SD = 0.83). That is, older people in the study indicated that social relationships for the usability of the house were more important than physical usability or space usability. For the HOOP questionnaire, the highest score corresponds to satisfaction with the physical characteristics of the dwelling (mean = 4.12 and SD = 0.90) more than with the security of the home or the propensity to change. For the MOH questionnaire, the factor with the highest mean was the emotional factor (mean = 4.30 and SD = 0.69), followed by the social factor (mean = 4.21 and SD = 0.73). That is, the meaning older people give to their home is related to emotional and social aspects more than cognitive, physical or behavioral aspects of the home. Finally, regarding the HCB questionnaire, internal control obtained the highest score (mean = 3.99 and SD = 0.63). Therefore, the HCB depend on older people and not on what other people or surroundings decide about the house.

### 3.3. Correlation between Domains of PH

To analyze the relationships between the domains of PH, as normality assumptions were not meet, we performed an analysis of Spearman’s rank correlations between the different habitat variables. Table 4 shows the main results of the correlations between variables. Significant positive correlations were observed between all the variables. The most significant correlations are between HOOP and UIMH (r = 0.684 **, *p* = 0.000), a greater satisfaction with the dwelling and a greater perception of usability. In addition, there is a high positive correlation between the MOH and HOOP, which indicates that a greater meaning of home is associated with better satisfaction with housing (r = 0.579 **, *p* = 0.000). Following the high correlation between the MOH and UIMH, a greater meaning of home was associated with a better perception of usability (r = 0.558 **, *p* = 0.000). There is also a positive correlation between HCB and the HOOP (r = 0.357 **, *p* = 0.000), between HCB and the UIMH (r = 0.363 **, *p* = 0.000), and between HCB and the MOH (r = 0.519 **, *p* = 0.000).

### 3.4. Estructural Equation Model

To analyze how the domains of PH interrelate and explains the RS of the active older adult, a structural equation model was used.

Structural equation modeling was performed to evaluate the adjustment between observed covariance structures and to develop a theoretical model of the effects between the latent variables. A latent variable is a variable that underlies a set of observed variables which shares the same explanatory construct. Structural equation modeling enables the representation and analysis of these covariance structures by means of the maximum likelihood method.

**Figure 1:**
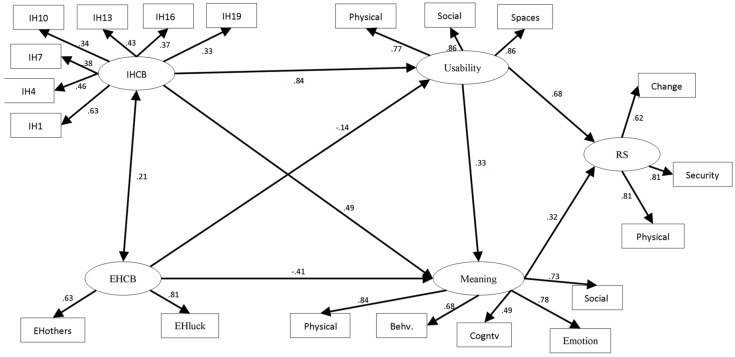
Structural equation model of internal housing-related control beliefs (IHCB), external housing-related control beliefs (EHCB), usability (Usability), meaning of home (Meaning) and housing options satisfaction (RS).

Through this analytical procedure, we will explore the dependency and association relationships that are established between the different evaluated dimensions of the PH of older people, as shown in Figure 1. This analysis included the exogenous latent variables, predictors of the perception of older people on internal housing-related control beliefs and their external housing-related control beliefs. These predicting variables are termed in the model “IHCB” and “EHCB”. Endogenous variables were the usability of the home (termed “usability”) and the meaning of the house (termed “meaning”), and finally, “RS” was used as a dependent variable.

Table 5 shows that all latent factors in the model are significantly related. The analyzed structural equation model obtained significant results for the different effect patterns.

Finally, Table 6 shows that there is a high adjustment of this model of relationships between HCB, usability, meaning and RS.

Taken together, we observe that IHCB over housing directly influences the usability (beta = 0.84) and perceived meanings of the home (beta = 0.49). However, the EHCB factors negatively influences the meaning of home (beta = −0.14). Additionally, a high perception of the usability of the residential habitat improves the meaning of the home space (beta = 0.33). Both the usability and the perceived meaning of home positively influence RS. However, the usability of housing has a greater weight on satisfaction (beta = 0.68) than its meaning (beta = 0.32).

## 4. Discussion

The RS is built on the perceived experiences about their home, and the objective aspects of the house or its environment are not the determining factors. The empirical model of PH allows us to incorporate these psychological elements into the study of the fit between older adults and their home environment and to better understand how RS is built [7,18]. The results obtained in our study show a high perceived RS regardless of gender or age group. This satisfaction is reflected in the physical characteristics of the house, in the perception of safety to develop activities in the house and in the PH change due to the aging process. Older adults are not resistant to change, but quite the opposite; it is important to consider that in the future, their current home may not be the best home solution, and they should be receptive to other proposals that allow them to continue their lifestyle. These data are consistent with the studies of Oswald [17] and Oswald & Muller [18], in which they found high perceptions not only in RS but also in domains of PH.

Oswald [17] showed that RS is interrelated with other domains of PH. Thus, RS is intimately linked to the perception of functionality, meaning and control. The different PHs are interconnected, weaving a model of relationships in which improvements in some perception lead to improvements in the other perceptions.

Beyond these relationships between the different PHs, the main objective of this study was to propose a model of the role that plays each of these perceptions in the formation of RS. According to our hypothesis, the model of structural equations shows that perceived RS is not another element within the PH but develops directly from perceptions of functionality and meaning. The functionality of the house brings environmental, personal and activity components to RS [20]. The data allow us to observe once again that those perceptions about the material and physical aspects of housing (environmental component), even if they play an important role in satisfaction, are not the only ones. The motivational aspects, the adaptive ability to carry out actions within the home, and the possibility of hosting social relations in it play a major role in final satisfaction [2,25]. Similarly, perceptions of emotional significance, the possibility of establishing social relationships at home, and the ability to develop everyday activities are also factors that increase RS.

Different studies [34,35] show how perceptions of meaning and functionality are an important factor in fit-e. In this study, we can also observe these perceptions in an integrative model, which allows us to see that functionality and meaning play a role in mediating variables between RS and perceived control.

In this way, perceived HCB occupies a decisive position in the model of structural equations. The RS depends on the type of perceived control over their home. To understand the effects of control perception on RS formation, it is necessary to segregate HCB from external housing-related control beliefs (EHCB) and internal housing-related control beliefs (IHCB), since both have opposite effects on the formation of RS. Increases in IHCB improve the mediating perceptions of functionality and meaning that end up generating RS. When older adults perceive that they control the tension between the demands of the environment and what can be done at home, they feel competent, and they generate an increase in the functionality and meaning of these tensions. The main effect of IHCB is on the perception of functionality, that is, the activities that can be done at home. Older people with a greater sense of competence will make more decisions about what they can do with their home, will be able to engage in more activities in the home, and will have an increased perception of the home’s functionality. Another effect of IHCB is on the meaning of home. Feeling competent about the set of experiences they may develop in the home increases affective, cognitive, and social aspects. Feeling competent and having control over the home is important since in the aging process, older people make the home the place of participation and social relations and they give it greater significance [36,37].

The opposite behavior is that generated by EHCB in the structural equation model. When decision-making about the home depends on external factors or other people who are beyond the control of older adults, i.e., other perceptions, RS deteriorates.

In summary, considering perceptions of meaning and usability, RS itself depends on a strong IHCB. Internal control influences everyday activities, and how social relationships are organized at home. When there is a high EHCB, either because decision-making about it is part of the social environment or because of chance, other perceptions deteriorate and therefore RS itself deteriorates.

Based on a high IHCB, older adults are ready for new home solutions. The largest respondents, despite expressing very high rates of satisfaction with the house itself, show a high propensity for change. They are open to changing their home and are open to new housing models in that they perceive that in a few years, they may lose control and autonomy in daily life [2]. Older adults doubt whether the conditions of the house itself will allow them to develop an autonomous and independent life as their aging process begins to diminish their functional capacities. These new home solutions must start from the basis that older adults are proactive people who participate in their immediate environments in which social relations are prioritized to establish new modes of home life. IHCB predisposes individuals to change as long as they continue to maintain control and autonomy over their lives [8,22].

The HCB is the result of the tension between a person’s competencies and the demands of the environment [26]. Social services could have an important role to play in promoting the IHCB of older people. The intervention should consider the environmental press that converges in the home and reduce this tension. To carry out this task, it is essential to know not only the dwellings conditions of the house but also the beliefs, expectations and subjective perceptions of older people about their home. As a possible result, we would not only improve housing adaptation, but aspects of physical and mental health and well-being can be enhanced. This statement needs further research to provide empirical evidence [2].

In future research, it would be interesting to analyze PH conditions and their relationship with other variables, such as social support and participation, life satisfaction and/or quality of life. Additionally, in future studies, we should compare the perceptions of older adults living in their own home with those who live in protected residences or flats whose autonomy is more limited. Finally, it would be interesting to conduct research on the four domains studied, functionality, satisfaction, meaning and control over the house itself in older people who are already living in other cohabitation models.

Of course, these findings are subject to limitations. This limitation may be seen in the generalization of the findings in old age. Although we analyzed data of a wide and heterogenous sample (rural–urban, alone–accompanied, etc.), the findings are based on an active older adult sample who attend Third Age universities, educational centers and senior associations. Thus, the present study is limited in its potential to reflect the full range of older adults with chronic diseases or disabilities, non-active older adults or older adults living in institutional settings. Another limitation is a significant gender gap in the sample, which could introduce gender bias in our finding.

## 5. Conclusions

Home is seen as the center of personal and social life, a basic element of participation in people’s most immediate environment. From the home itself, people perceive ample possibilities of social interaction and of maintaining a rich and complex social life that allows them to develop their own personality and identity [38]. These elements of social participation promote active aging [39] and are complexly related to benefits in both physical and mental health status [2].

Home proposals that lead to a deterioration in the perception of control over the house itself can have negative effects on active older adults. For example, it could be the case of housing solutions that institutionalize the older adult, who loses control over their own home and reduces their autonomous action. This type of proposal focuses on disease monitoring and care rather than encouraging independent participation of older adults. Thus, if the models generated from the data are successful, we can predict a clear decrease in the RS of people associated with this sense of loss of control, which leads to the formation of perceptions about the habitat itself that are not meaningful and functional. Providing a sustainable home alternative based not on the age-related deterioration model but on the capacity for interaction and social participation of older people and on their ability to contribute and return to society and preserve their set of treasured experiences should be the approach to designing sustainable home solutions.

## Figures and Tables

**Table 1:** Sociodemographic characteristics of the sample.

Sociodemographic Characteristics	Number	%
Sex		
Male	194	27.6
Female	508	72.4
Education		
Without studies	288	38.5
Primary studies	377	54.1
Average or higher studies	52	7.5
Age		
From 50 to 59 years	74	10.7
From 60 to 69 years	344	49.6
From 70 to 79 years	218	31.5
80 years to more	57	8.2
Coexistence		
Alone	160	22.8
Accompanied	543	77.2
Householder		
Rented	36	5.3
Owned	546	94.7
House size		
<90 m^2^	184	32.7
90–120 m^2^	170	30.2
>120 m^2^	209	37.1
Location		
Urban	395	55.3
Rural	319	44.68

**Table 2:** Mean and standard deviation of scale of the domains of PH and gender.

Scales	Mean (SD)	Male (SD)	Female (SD)
Usability in My Home	4.22 (0.76)	4.07 (0.074)	4.31 (0.76)
Housing Options for Older People	4.06 (0.78)	3.92 (0.77)	4.02 (0.81)
The Meaning of Home	3.69 (0.46)	4.03 (0.50)	4.10 (0.55)
Housing-related control beliefs (HCB)	3.20 (0.57)	3.07(0.61)	3.44 (0.51)

**Table 3:** Mean and standard deviation of the factors of each scale.

Scales	Mean	SD
HOOP		
Physical	4.1201	0.90186
Security	4.0156	0.84383
Propensity change	3.7458	1.11200
UIMH		
Physical	4.3113	0.83940
Social	4.3422	0.83368
Spaces	4.1228	0.85919
MOH		
Physical	4.1492	0.67694
Behavioral	4.0478	0.64943
Cognitive	3.7324	0.72273
Emotional	4.3094	0.69397
Social	4.2158	0.73294
HCB		
Internal	3.9903	0.63420
External Others	2.8214	0.74470
External luck	2.8897	0.91323
External	2.85	0.71000

**Table 4:** Correlation analyses between the scales.

	HOOP	UIMH	MOH	HCB
HOOP	1	0.684 **	0.579 **	0.357 **
UIMH		1	0.558 **	0.363 **
MOH			1	0.519 **
HCB				1

** Significance correlation at level 0.01 (bilateral). * Significance correlation at level 0.05(bilateral).

**Table 5:** Structural effect parameters.

	Coef.	OIM Std. Err.	Z	P ˃ Z	[95% Conf. Interval]	
Usability						
IHCB	0.8434602	0.0338105	24.95	0.000	0.7771928	0.9097275
EHCB	−0.130863	0.046903	−2.90	0.000	−0.2280145	−0.0441581
Meaning						
Usability	0.3274796	0.1007887	3.25	0.001	0.1299373	0.5250219
IHCB	0.4923202	0.1100427	4.47	0.000	0.2766404	0.7079999
EHCB	−0.4140296	0.0473664	−8.74	0.000	−0.506866	−0.3211932
RS						
Usability	0.6838675	0.0404377	16.91	0.000	0.604611	0.763124
Meaning	0.3197726	0.3197726	7.26	0.000	0.233921	0.406153

**Table 6:** Goodness-of-fit indices of the model.

Indices	Observed Values	Suitable Values
χ^2^	539.79 (g.l. = 0.269, *p* < 0.000)	Better when smallest (*p* not significant)
CFI	0.92 *	Greater than 0.90; better when nearer to 1
TLI	0.91 *	Between 0.90 and 1 indicates a good fit
RMSEA	0.05 **	Equal or less than 0.05 indicates a good fit

* Values nearest to 1 indicates a good fit. ** Values nearest to 0 indicates a better good fit of the model.

## Data Availability

The raw data supporting the conclusions of this article will be made available by the authors, without undue reservation, to any qualified researcher.

## References

[1] Baltes M.M., Maas I., Wilms H.-U., Borchelt M., Little T.D., Baltes P.B., Mayer K.U. (1998). Everyday Competence in Old and Very Old Age: Theoretical Considera-tions and Empirical Findings.

[2] Iwarsson S., Löfqvist C., Oswald F., Slaug B., Schmidt S., Wahl H.-W., Tomsone S., Himmelsbach I., Haak M. (2016). Synthesizing ENABLE-AGE Research Findings to Suggest Evidence-Based Home and Health Interventions. J. Hous. Elder..

[3] Oswald F., Wahl H.-W., Schilling O., Nygren C., Fänge A.M., Sixsmith A., Sixsmith J., Szeman Z., Tomsone S., Iwarsson S. (2007). Relationships between Housing and Healthy Aging in Very Old Age. Gerontologist.

[4] Wahl H.-W., Iwarsson S., Oswald F. (2012). Aging Well and the Environment: Toward an Integrative Model and Research Agenda for the Future. Gerontologist.

[5] Wahl H.-W., Schilling O., Oswald F., Iwarsson S. (2009). The home environment and quality of life-related outcomes in advanced old age: Findings of the ENABLE-AGE project. Eur. J. Ageing.

[6] Gefenaite G., Björk J., Schmidt S.M., Slaug B., Iwarsson S. (2019). Associations among housing accessibility, housing-related control beliefs and independence in activities of daily living: A cross-sectional study among younger old in Sweden. N. J. Hous. Environ. Res..

[7] Drewelies J., Wagner J., Tesch-Römer C., Heckhausen J., Gerstorf D. (2017). Perceived control across the second half of life: The role of physical health and social integration. Psychol. Aging.

[8] Oswald F., Wahl H.W., Rowles G.D., Bernard M. (2013). Creating and Sustaining Homelike Places in Own Home Environments.

[9] Oswald F., Wahl H.-W. (2019). Physical Contexts and Behavioral Aging.

[10] Pettersson C., Slaug B., Granbom M., Kylberg M., Iwarsson S. (2018). Housing accessibility for senior citizens in Sweden: Estimation of the effects of targeted elimination of environmental barriers. Scand. J. Occup. Ther..

[11] Slaug B., Granbom M., Iwarsson S. (2020). An Aging Population and an Aging Housing stock—Housing Accessibility Problems in Typical Swedish Dwellings. J. Aging Environ..

[12] Kylén M., Schmidt S., Iwarsson S., Haak M., Ekström H. (2017). Perceived home is associated with psychological well-being in a cohort aged 67–70 years. J. Environ. Psychol..

[13] Clark A., Rowles G., Bernard M. (2013). Environmental Gerontology: Making Meaningful Places in Old Age.

[14] Iwarsson S., Wahl H.-W., Nygren C., Oswald F., Sixsmith A., Sixsmith J., Szeman Z., Tomsone S. (2007). Importance of the Home Environment for Healthy Aging: Conceptual and Methodological Background of the European ENABLE–AGE Project. Gerontol..

[15] Oswald F., Wahl H.W., Rowles G.D., Chaudhury H. (2005). Dimensions of the meaning of home. Home and Identity in Later Life. International Perspectives.

[16] Heywood F. (2005). Adaptation: Altering the House to Restore the Home. Hous. Stud..

[17] Oswald F., Schilling O., Wahl H.-W., Fänge A., Sixsmith J., Iwarsson S. (2006). Homeward bound: Introducing a four-domain model of perceived housing in very old age. J. Environ. Psychol..

[18] Oswald F., Kašpar R. (2012). On the Quantitative Assessment of Perceived Housing in Later Life. J. Hous. Elder..

[19] Müller H., Oswald F. (2020). An Intergenerational Approach to Perceived Housing. J. Aging Environ..

[20] Fänge A.M., Iwarsson S. (2005). Changes in ADL Dependence and Aspects of Usability Following Housing Adaptation--A Longitudinal Perspective. Am. J. Occup. Ther..

[21] Fänge A., Iwarsson S. (2005). Changes in accessibility and usability in housing: An exploration of the housing adaptation process. Occup. Ther. Int..

[22] Oswald F., Wahl H.-W., Martin M., Mollenkopf H. (2003). Toward measuring proactivity in person-environment transactions in late adulthood: The housing-related control beliefs questionnaire. J. Hous Elder..

[23] Parmelee P.A., Lawton M.P., Birren J.E., Schaie K.W. (1990). The design of special environments for the aged. Handbook of the Psychology of Aging.

[24] Iwarsson S., Wahl H.-W., Nygren C. (2004). Challenges of cross-national housing research with older persons: Lessons from the EN-ABLE-AGE project. E. J. Ageing..

[25] Martins P., Ornelas J., Silva A.C. (2016). The role of perceived housing quality and perceived choice to recovery: An ecological per-spective on a housing first program in Lisbon. J. Environ. Psychol..

[26] Lawton M.P., Nahemow L., Eisdorfer C., Lawton M.P. (2004). Ecology and the aging process. The Psychology of Adult Development and Aging.

[27] Fänge A., Iwarsson S. (1999). Physical housing environment: Development of a self-assessment instrument. Can. J. Occup. Ther..

[28] Iwarsson S. (2005). A Long-Term Perspective on Person-Environment Fit and ADL Dependence among Older Swedish Adults. Gerontologist.

[29] Angelini V., Bucciol A., Wakefield M., Weber G. (2019). Can temptation explain housing choices over the life cycle?. Manch. Sch..

[30] Borsch-Supan A., Kneip T., Litwin H., Myck M., Weber G. (2015). Ageing in Europe—Supporting Policies for an Inclusive Society.

[31] Heywood F., Oldman C., Means R. (2002). Housing and Home in Later Life.

[32] Boonyaratana Y., Hansson E.E., Granbom M., Schmidt S.M. (2021). The psychometric properties of the Meaning of home and hous-ing-related control beliefs scales among 67–70 year-Olds in Sweden. Int. J. Environ. Res. Public Health.

[33] Andersson N., Nilsson M.H., Slaug B., Oswald F., Iwarsson S. (2020). The meaning of home questionnaire revisited: Psychometric analyses among people with Parkinson’s disease reveals new dimensions. PLoS ONE.

[34] Dahlin-Ivanoff S., Haak M., Fänge A.M., Iwarsson S. (2007). The multiple meaning of home as experienced by very old Swedish people. Scand. J. Occup. Ther..

[35] Haak M., Fänge A.M., Horstmann V., Iwarsson S. (2008). Two dimensions of participation in very old age and their relations to home and neighborhood environments. Am. J. Occup. Ther..

[36] Haak M., Fänge A.M., Iwarsson S., Ivanoff S.D. (2007). Home as a signification of independence and autonomy: Experiences among very old Swedish people. Scand. J. Occup. Ther..

[37] Gefenaite G., Björk J., Iwarsson S., Slaug B., Schmidt S.M., Nilsson M.H. (2020). Longitudinal association between housing accessibility and activities of daily living: The role of self-efficacy and control in people ageing with Parkinson’s disease. BMC Geriatr..

[38] Iwarsson S., Horstmann V., Carlsson G., Oswald F., Wahl H.-W. (2009). Person—Environment fit predicts falls in older adults better than the consideration of environmental hazards only. Clin. Rehabil..

[39] Horstmann V., Haak M., Tomsone S., Iwarsson S., Gräsbeck A. (2012). Life satisfaction in older women in Latvia and Sweden-relations to standard of living, aspects of health and coping behaviour. J. Cross Cult. Gerontol..

